# Prediction Analysis for Transition to Schizophrenia in Individuals at Clinical High Risk for Psychosis: The Relationship of *DAO, DAOA*, and *NRG1* Variants with Negative Symptoms and Cognitive Deficits

**DOI:** 10.3389/fpsyt.2017.00292

**Published:** 2017-12-20

**Authors:** Vinita Jagannath, Anastasia Theodoridou, Miriam Gerstenberg, Maurizia Franscini, Karsten Heekeren, Christoph U. Correll, Wulf Rössler, Edna Grünblatt, Susanne Walitza

**Affiliations:** ^1^Department of Child and Adolescent Psychiatry and Psychotherapy, University Hospital of Psychiatry Zurich, University of Zurich, Zurich, Switzerland; ^2^The Zurich Program for Sustainable Development of Mental Health Services (ZInEP), University Hospital of Psychiatry Zurich, Zurich, Switzerland; ^3^Department of Psychiatry, Psychotherapy and Psychosomatics, University Hospital of Psychiatry Zurich, University of Zurich, Zurich, Switzerland; ^4^The Zucker Hillside Hospital, Psychiatry Research, Northwell Health, Glen Oaks, NY, United States; ^5^Hofstra Northwell School of Medicine, Hempstead, NY, United States; ^6^The Feinstein Institute for Medical Research, Manhasset, NY, United States; ^7^Neuroscience Center Zurich, University of Zurich and ETH Zurich, Zurich, Switzerland; ^8^Zurich Center for Integrative Human Physiology, University of Zurich, Zurich, Switzerland

**Keywords:** d-amino acid oxidase/DAO/DAAO, d-amino acid oxidase activator/G72/DAOA, neuregulin 1/NRG1, attenuated positive symptoms syndrome/APSS, single-nucleotide polymorphism/SNP, research domain criteria/RDoC

## Abstract

Schizophrenia is characterized by positive and negative symptoms and cognitive dysfunction. The glutamate hypothesis of schizophrenia has been hypothesized to explain the negative symptoms and cognitive deficits better than the dopamine hypothesis alone. Therefore, we aimed to evaluate whether glutamatergic variants such as d-amino acid oxidase (*DAO*), DAO activator (*DAOA*)/*G72*, and neuregulin 1 (*NRG1*) single-nucleotide polymorphisms (SNPs) and their mRNA levels predicted (i) transition to schizophrenia spectrum disorders and (ii) research domain criteria (RDoC) domains, mainly negative valence and cognitive systems. In a 3-year prospective study cohort of 185 individuals (age: 13–35 years) at high risk and ultra-high risk (UHR) for psychosis, we assessed *DAO* (rs3918347, rs4623951), *DAOA* (rs778293, rs3916971, rs746187), and *NRG1* (rs10503929) SNPs and their mRNA expression. Furthermore, we investigated their association with RDoC domains, mainly negative valence (e.g., anxiety, hopelessness) and cognitive (e.g., perception disturbances, disorganized symptoms) systems. *NRG1* rs10503929 CC + CT versus TT genotype carriers experienced significantly more disorganized symptoms. *DAOA* rs746187 CC versus CT + TT genotype, *DAOA* rs3916971 TT versus TC + CC genotype, and *DAO* rs3918347 GA + AA versus GG genotype carriers experienced nominally more hopelessness, visual perception disturbances, and auditory perception disturbances, respectively. The schizophrenia risk G-allele of *DAO* rs3918347 nominally increased risk for those UHR individuals with attenuated positive symptoms syndrome. No association between *DAO, DAOA, NRG1* SNPs, and conversion to schizophrenia spectrum disorders was observed. Our findings suggest that *DAO, DAOA*, and *NRG1* polymorphisms might influence both RDoC negative valence and cognitive systems, but not transition to schizophrenia spectrum disorders.

## Introduction

Schizophrenia is a chronic and debilitating disorder, preceded by a broad range of symptoms. The emergence of psychotic features in schizophrenia is typically between the late teens and mid-30s (1). Early recognition of individuals at-risk for psychosis and the provision of early intervention is likely to be associated with improved outcomes (2). Individuals at clinical risk for psychosis are identified by two complementary approaches: the high risk (HR) and the ultra-high risk (UHR) criteria. The HR concept is based on basic symptoms and comprises two partially overlapping risk constellations, the cognitive-perceptive (COPER) basic symptoms and the cognitive disturbances (COGDIS) (3, 4). The UHR criteria comprise attenuated positive symptoms syndrome (APSS), brief limited intermittent psychotic symptoms (BLIPS), and a combination of a risk factor for psychosis and a recent functional decline (5). A meta-analysis of 27 studies showed that 18, 21, 27, and 32% of individuals at-risk for psychosis (HR + UHR) transitioned to psychotic disorders at 6, 12, 24, and 36 months of follow-up, respectively (6). This meta-analysis also showed that the mean transition risk was 49, 28, and 22% using the HR approach, UHR approach, and when combining both HR and UHR approaches, respectively (6). One study reported that about one-third of individuals at UHR for psychosis transitioned to psychosis (7). Our recent study, using a multivariable prediction model, demonstrated that as expected, UHR criteria predicted conversion to psychosis but combining HR and UHR criteria in this help-seeking at-risk population did not improve the predictive accuracy of UHR alone (8). Therefore, it is important to optimize the identification of individuals at HR/UHR for psychosis by minimizing the false positive rate and improving the true positive prediction rate of conversion to psychosis.

The estimated heritability in schizophrenia is around 60–80% (9). Studies have demonstrated an association between schizophrenia and d-amino acid oxidase (*DAO*), DAO activator (*DAOA*)/G72, and neuregulin 1 (*NRG1*) single-nucleotide polymorphisms (SNPs) (10, 11). *DAOA* and *NRG1* polymorphisms were shown to predict the transition to schizophrenia in individuals at HR/UHR for psychosis (12–14). Genetic studies in schizophrenia have shown the association of neurocognitive endophenotypes with several glutamatergic gene polymorphisms including *NRG1* (15–17). These studies suggest that genetic variations and neurocognitive endophenotypes may help to improve the prediction accuracy of clinical symptoms and HR/UHR criteria for transition in an at-risk population.

The glutamate hypothesis of schizophrenia originated from the observation that *N*-methyl-d-aspartate (NMDA) receptor blockers like ketamine induced schizophrenia-like symptoms. As antipsychotics (dopamine D2 receptor antagonists) have little effect on negative symptoms and cognitive deficits, the glutamatergic system is an attractive therapeutic target (18). Meta-analyses have reported that addition of NMDA receptor agonist d-serine and glycine transporter type 1 inhibitor sarcosine as an adjunct to antipsychotics reduce total and negative symptoms (19, 20). Based on these observations, the glutamate hypothesis is thought to describe the pathophysiology underlying negative symptoms and cognitive deficits better than the dopamine hypothesis (21–23). NMDA receptor hypofunction might lead to decreased dopamine activity in the mesocortical pathway, which may manifest as negative symptoms and cognitive dysfunction in schizophrenia (24). The NMDA receptor hypofunction theory proposed in schizophrenia might be partly explained by increased DAO activity modulated by DAOA leading to decreased d-serine, a co-agonist of NMDA receptors (25). The function of NRG1 is mediated by binding to receptor tyrosine kinases called ErbB (ErbB3 and ErbB4), and an altered NRG1/ErbB4 signaling is thought to result in NMDA receptor hypofunction (26, 27). These studies highlight the potential pathogenic link between NMDA receptor hypofunction and dysregulation of *DAO, DAOA*, and *NRG1* genes.

The National Institute of Mental Health started the research domain criteria (RDoC) initiative to guide and organize research in psychiatric disorders beyond the typical diagnostic classification approach (28). This initiative provides a non-disease-based structured conceptual framework to understand the dimensional range of human behavior from normal to abnormal by integrating multiple levels of information (from genomics to self-reports). The RDoC represents a paradigm shift from Diagnostic and Statistical Manual of Mental Disorders (DSM)/International Classification of Diseases (ICD) to dimensional approaches with an aim to integrate basic research and psychopathology (29).

In this study, we aimed (1) to identify predictive glutamatergic genetic polymorphisms in at-risk individuals for transition to schizophrenia spectrum disorders, (2) to identify endophenotypes potentially linked to the glutamatergic system in at-risk individuals using RDoC constructs, and (3) to evaluate differences in *DAO, DAOA*, and *NRG1* mRNA levels across clinical and RDoC domains.

## Materials and Methods

### Study Population

Participants were recruited by the “Early Recognition and Intervention Program for Psychosis and Bipolar Disorder” project as part of the Zurich Program for Sustainable Development of Mental Health Services (ZInEP)^1^ in the Canton of Zurich, Switzerland. The detailed design, inclusion, and exclusion criteria of the study were described in our previous studies (30, 31). A total of 185 individuals aged 13–35 years at HR/UHR for psychosis were assessed at baseline and were subsequently followed-up at 6, 12, 24, and 36 months for the transition to schizophrenia. At 36 months follow-up, 50% of individuals (*n* = 93) dropped out of the study. The dropouts were due to refusal to participate in the study in most of the cases and due to non-response after contacting in few cases. However, we are unable to give exact numbers of individuals who refused to participate or who did not respond due to missing information in several study participants. Individuals at HR for psychosis were assessed using the Schizophrenia Proneness Instrument-Child and Youth version (SPI-CY) (age < 18 years) (32, 33) or Schizophrenia Proneness Instrument-Adult version (SPI-A) (age ≥ 18 years) (34) and were included when they had one COPER basic symptom or at least two COGDIS. Individuals at UHR for psychosis were assessed using the Structured Interview for Prodromal Syndromes (SIPS) (35, 36) and were included when they met criteria for the APSS or the BLIPS or the state-trait criteria (>30% reduction in global assessment of functioning in the past year plus either schizotypal personality disorder or a first-degree relative with psychosis). The transition to schizophrenia was defined according to ICD-10 criteria (37). The severity of positive and negative symptoms was assessed using the Positive and Negative Syndrome Scale (PANSS) (38), severity of depressive symptoms with the Calgary Depression Rating scale for Schizophrenia (CDSS) (39), and anxiety symptoms with the Beck Anxiety Inventory (BAI) (40). The demographic and diagnostic characteristics of the study population are shown in Table S1 in Supplementary Material. This study was approved by the Cantonal Ethics Commission of Zurich (Ref. Nr. EK: E-63/2009) and complies with the Declaration of Helsinki. Informed written consent was obtained from adult participants and legal guardians of minors, and written assent was obtained from minors.

### Phenotypic Domains

The participants were grouped into clinical phenotypes (cases versus controls) namely, converters to schizophrenia spectrum disorders (*n* = 27), i.e., schizophrenia, schizophreniform disorder, and acute psychotic disorder, versus non-converters (*n* = 65) at 36 months follow-up [dropouts (*n* = 93)], and APSS (*n* = 98) group versus all other help-seeking individuals (BLIPS, state-trait criteria, COGDIS, COPER; *n* = 87) at baseline. The transition to schizophrenia was defined according to ICD-10 criteria (37).

First, we grouped our cohort as per the factor structure of the PANSS, concentrating on negative symptoms and general psychopathology (38). As we did not find any significant differences in negative symptoms (sum score of negative (N1–N7) PANSS subscale) and general psychopathology (sum score of general psychopathology (G1–G16) PANSS subscale) across *DAO, DAOA*, and *NRG1* SNPs, we then decided to use an exploratory approach by focusing on subgroups of psychopathology constructs defined according to the RDoC domains. The RDoC framework consists of five domains namely, negative valence systems, positive valence systems, cognitive systems, systems for social process, and arousal/regulatory systems (41). In this study, we decided to concentrate on two RDoC domains: negative valence systems and cognitive systems, due to the potential role of *DAO, DAOA*, and *NRG1* polymorphisms in the glutamate hypothesis of schizophrenia, as it appears to explain the pathogenesis of negative symptoms and cognitive deficits better than the dopamine hypothesis (22, 23, 42) and unavailability of relevant neuropsychological scales in our study to create the positive valence domain. Negative valence systems focus on responses to aversive situations, such as fear, anxiety, and loss. Cognitive systems concentrate on various cognitive processes, such as perception, language, memory, and cognitive control. Within negative valence systems, we focused on the constructs of threat (acute and sustained) and loss. The negative valence system construct of threat was assessed by the total score of BAI with higher BAI scores pointing to increased severity of anxiety (40). The negative valence system construct loss was assessed by the response to the CDSS item 2 “hopelessness,” which can be scored as 0 (absent), 1 (mild), 2 (moderate), or 3 (severe) (39). Within cognitive systems, we chose the constructs of perception (visual and auditory) and cognitive control. The cognitive system construct visual perception was assessed by summing the following four SPI-A (and equivalent SPI-CY) items O4 (other visual perception disturbances), F1 (hypersensitivity to light), F2 (photopsia), and F3 (micropsia/macropsia), with higher scores pointing to more frequent disturbances in visual perception. The cognitive system construct of auditory perception was assessed by summing the following three SPI-A (and equivalent SPI-CY) items O5 (other acoustic perception disturbances), F4 (hypersensitivity to sounds/noise), and F5 (changed intensity/quality of acoustic stimuli), with higher scores pointing to more frequent disturbances in auditory perception. The cognitive system construct of cognitive control was obtained by summing the following four SIPS disorganization items D1, D2, D3, and D4 (35, 36), with higher scores indicating more severe disturbance in disorganized symptoms.

The above-mentioned scales used to tap into RDoC domains were assessed at baseline and last-available follow-up until 36 months. As there was a dropout rate of 50% at 36 months, if there were no data available at 36 months, we took the scores from the last follow-up that the individual attended (i.e., 6 or 12 or 24 months).

### TaqMan SNP Genotyping

The study population was genotyped for *DAO* (rs3918347, rs4623951), *DAOA* (rs778293, rs3916971, rs746187), and *NRG1* (rs10503929) SNPs. These SNPs were selected based on previously reported significant association with schizophrenia (10, 43). In our recent meta-analysis, we found a significant association of *DAO* rs4623951 [odds ratio (OR) = 0.88; minor allele: C], *DAOA* rs778293 (OR = 1.17; minor allele: G), *DAOA* rs3916971 (OR = 0.84; minor allele: T), and *NRG1* rs10503929 (OR = 0.89; minor allele: C) with schizophrenia (11). In this study, the carriers of the risk allele of *DAO, DAOA*, and *NRG1* SNPs were anticipated to have worse psychopathology scores in RDoC-negative valence and cognitive system constructs. DNA was isolated from whole blood ethylenediaminetetraacetic acid tubes collected from the study population using QIAamp DNA Blood Maxi Kit (Qiagen) as per manufacturer’s protocol. A spectrophotometer (NanoVue Plus, GE) was used to measure DNA concentrations, A260/A280, and A260/A230 ratios. The study population was genotyped for *DAO* (rs3918347 assay number: C_27937201_10, rs4623951 assay number: C_32177440_10, both from Applied Biosystems, USA), *DAOA* (rs778293 assay number: C_8704507_10, rs3916971 assay number: C_27495752_10, rs746187 assay number: C_1925241_10, all from Applied Biosystems, USA), and *NRG1* (rs10503929, assay number: C_2870393_10, Applied Biosystems, USA) SNPs (44). DNA (10 ng/µl); TaqMan^®^ Genotyping Master Mix (Applied Biosystems, USA); and above-mentioned *DAO, DAOA*, and *NRG1* SNP Genotyping Assays (Applied Biosystems, USA) were combined in a 384-well plate. Real-time polymerase chain reaction (PCR) was performed in a C1000™CFX384™ Thermal cycler (Bio-Rad) using TaqMan^®^ SNP Genotyping Assay PCR standard protocol. The allelic discrimination program of Bio-Rad CFX Manager™ Software version 2.1 was used to determine genotypes (44). Samples were run in duplicates to ensure correct results. In case of ambiguity in duplicates, genotyping was repeated in a separate run to ensure correct results. No-template controls were included in every run to exclude impurities.

### Quantification of *NRG1, DAO*, and *DAOA* mRNA Levels Using Quantitative Real-time Reverse Transcription-Polymerase Chain Reaction (qRT-PCR)

RNA was isolated from whole blood collected from the study population using PAXgene Blood RNA Kit (Qiagen) according to manufacturer’s protocol. A spectrophotometer (NanoVue Plus, GE) was used to measure RNA concentrations, A260/A280, and A260/A230 ratios. RNA integrity was analyzed using Experion automated electrophoresis system (Bio-Rad) in a subset of samples to ensure RNA integrity number/RNA quality indicator >7. RNA (500 ng) was reverse transcribed using iScript™ cDNA Synthesis Kit (Bio-Rad) as per manufacturer’s protocol. In a subset of samples, negative controls were prepared with RNA using iScript™ cDNA Synthesis Kit (Bio-Rad) without reverse transcriptase enzyme as per manufacturer’s protocol. qRT-PCR was performed using cDNA, QuantiFast SYBR Green PCR kit (Qiagen), 1 µM *NRG1* primer (QT00061964, Qiagen), and reference genes [β-actin (*ACTB*) (QT01680476), aminolevulinate synthetase (*ALAS1*) (QT00073122), ribosomal protein L13a (*RPL13A*) (QT00089915), alanyl-tRNA synthetase (*AARS*) (QT00054747), glyceraldehyde-3-phosphate dehydrogenase (*GAPDH*) (QT01192646), peptidyl prolyl isomerase A (*PPIA*) (QT00866137), and X-prolyl aminopeptidase1 (*XPNPEP1*) (QT00051471); all from Qiagen]. *NRG1* mRNA levels were normalized to the references genes (44). PCR efficiencies were calculated using LinReg PCR program (45), and mean PCR efficiencies for all studied amplicons were found to be between 91 and 93%. Normalized *NRG1* mRNA levels were quantified using qBASE plus software (Biogazelle), which utilizes gene-specific amplification efficiencies and allows normalization with multiple reference genes (46).

We performed qRT-PCR to detect *DAO* mRNA using *DAO* primers described by Verrall et al. (47) and predesigned primers [qHsaCID0011122 and qHsaCEP0058247 (Bio-Rad), Hs.PT.58.3248433 and Hs.PT.58.45768871 (IDT)]. We performed qRT-PCR to detect *DAOA* mRNA using primers for *DAOA* gene described by Benzel et al. (48), Cheng et al. (49), and pre-designed primers [QT00058863 (Qiagen), Hs.PT.58.555086 (IDT), 4331182 (ThermoFisher scientific), qHsaCEP0024792 (Bio-Rad)]. QuantiTect Whole Transcriptome Kit (207043, Qiagen) followed by qRT-PCR was also used to detect *DAO* and *DAOA* mRNA levels (44). However, we were unable to quantify *DAO* and *DAOA* mRNA with the aforementioned methods in the whole blood as either no signal was observed or genomic DNA was amplified concomitantly.

### Statistical Analysis

The results from SNP genotyping was analyzed using PLINK software (50). The minor allele frequency (MAF) and deviation from Hardy-Weinberg Equilibrium (HWE) was computed using PLINK software, and *p* < 0.05 was considered as statistically significant. The *DAO* (rs3918347), *DAOA* (rs3916971, rs778293, rs746187), and *NRG1* (rs10503929) SNPs were in HWE (*p* > 0.05), and the MAF of *DAO, DAOA*, and *NRG1* SNPs were similar to HapMap CEU MAF (Table S5 in Supplementary Material). The *DAO* rs4623951 SNP deviated from the HWE (*p* < 0.05; Table S4 in Supplementary Material). The differences in allele and genotype frequencies across clinical phenotypes (cases versus controls) were assessed using Chi-square test, and *p* < 0.05 was considered as statistically significant. The OR, 95% CI, and *p* value for SNP models (allelic, dominant, and recessive) across clinical phenotypes were calculated from allele/genotype frequencies using an online OR calculator,^2^ and *p* value was adjusted based on the number of analyzed SNPs (Bonferroni correction, *p* < 0.008). The *post hoc* power analyses for association of *DAO, DAOA*, and *NRG1* SNPs with converters to schizophrenia spectrum disorders versus non-converters (Table S5 in Supplementary Material) and APSS versus all other help-seeking group were conducted using Fisher’s exact test of independence in G*Power software (51), the calculated OR was used, and the alpha level was set at 0.05.

IBM^®^ SPSS^®^ Statistics (version 21) software was used for statistical analysis. Shapiro–Wilk test with Lilliefors significance correction was used to assess the normality of the distribution of *NRG1* gene expression and clinical scales (BAI, CDSS, SPI-A/SPI-CY, SIPS, and PANSS). *NRG1* gene expression and clinical scales showed both normal and non-normal distribution. We used non-parametric tests even for normally distributed data to maintain consistency between statistical evaluations. The differences in RDoC domains (negative valence and cognitive systems) across models (genotypic, dominant, recessive) were assessed using Mann–Whitney test (for two groups) or Kruskal–Wallis test (for >2 groups), and *p* values were adjusted based on the number of constructs analyzed (Bonferroni correction, *p* < 0.008). The differences in *NRG1* mRNA levels across clinical phenotypes were assessed using Mann–Whitney test, and *p* < 0.05 was set as statistically significant. The *post hoc* power analyses for RDoC-negative valence and cognitive systems across *DAO, DAOA*, and *NRG1* SNPs were conducted using analysis of variance test for three groups or *t*-test for two groups in G*Power software (51), the effect sizes were determined from means of neuropsychological scales used in RDoC-negative valence and cognitive systems, and the alpha level was set at 0.05 (Table S7 in Supplementary Material). The differences in *NRG1* mRNA levels across *NRG1* rs10503929 SNP genotypes was assessed using Kruskal–Wallis test, and across dominant (CC + CT, TT) and recessive models (CC, CT + TT) were assessed using Mann–Whitney test, with *p* < 0.05 being set as statistically significant. The correlation between *NRG1* gene expression and RDoC domains (negative valence and cognitive systems) was assessed using Spearman’s rank correlation test, and *p* < 0.05 was considered statistically significant.

## Results

The *DAO* (rs3918347), *DAOA* (rs3916971, rs778293, rs746187), and *NRG1* (rs10503929) SNPs were in HWE (*p* > 0.05), and the MAF of *DAO, DAOA*, and *NRG1* SNPs were similar to HapMap CEU MAF (Table S2 in Supplementary Material).

### *DAO, DAOA*, and *NRG1* Polymorphisms across Clinical Phenotypes

There were no significant associations between *DAO, DAOA*, and *NRG1* SNPs with converters to schizophrenia spectrum disorders compared to non-converters at 36 months follow-up (power range: 0.05–0.31; Table S3 in Supplementary Material). However, there was a nominal association (*p* > 0.008) of *DAO* rs3918347 with APSS compared to all other help-seeking group at baseline, and the G-allele had a tendency to be a risk allele for APSS (OR = 1.84, 95% CI = 1.13–3.01, *p* = 0.01), with a power of 0.76 (Table S4 in Supplementary Material). There were no significant associations between the rest of the *DAO, DAOA, NRG1* SNPs and APSS (power range: 0.05–0.89; Table S4 in Supplementary Material).

### *DAO, DAOA*, and *NRG1* Polymorphisms across RDoC Domains

**Figure 1:**
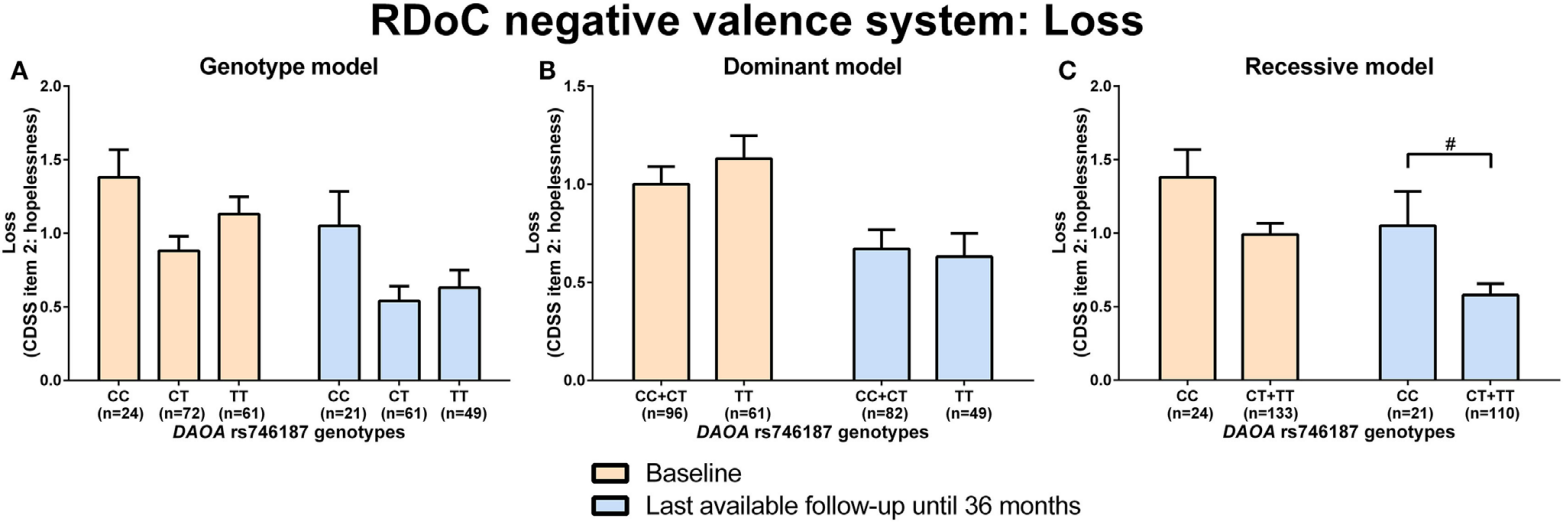
Differences in research domain criteria (RDoC) negative valence system loss across d-amino acid oxidase activator (*DAOA*) rs746187 genotypes. Differences in Calgary Depression Scale for Schizophrenia (CDSS) item 2: hopelessness scale across *DAOA* rs746187 genotypes in genotype **(A)**, dominant **(B)**, and recessive **(C)** model at baseline and last-available follow-up time point until 36 months. Data are presented as mean ± SEM; ^#^0.008 < *p* < 0.05 (significant without Bonferroni correction).

**Figure 2:**
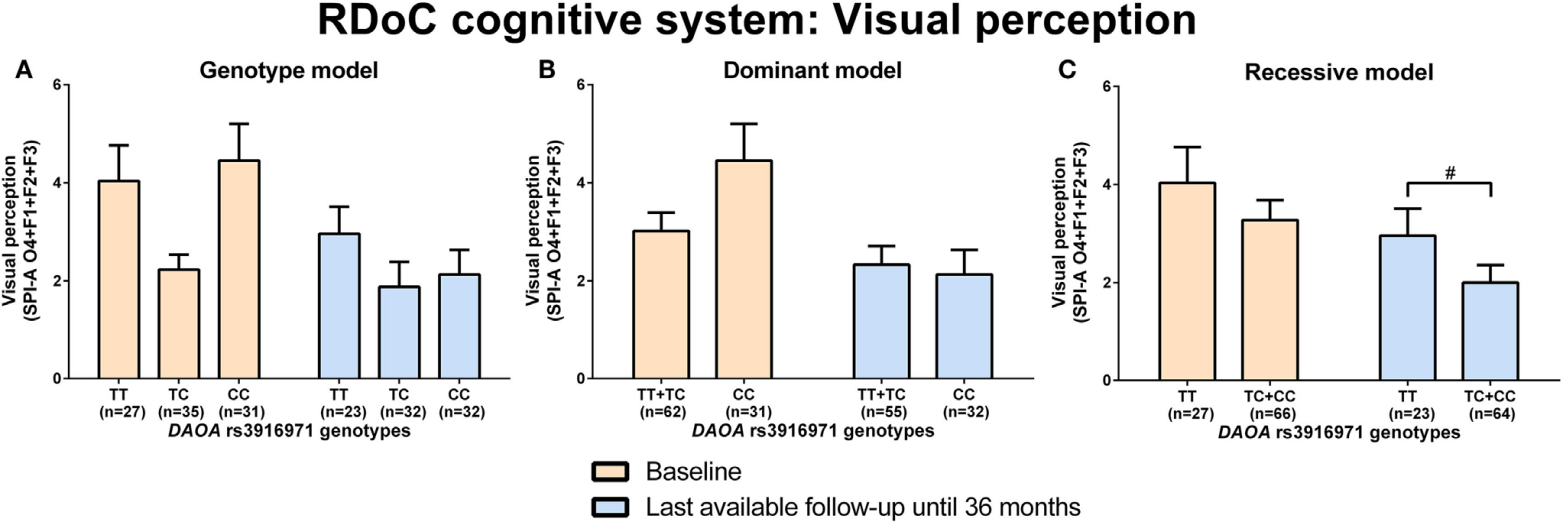
Differences in research domain criteria (RDoC) cognitive system visual perception across d-amino acid oxidase activator (*DAOA*) rs3916971 genotypes. Differences in Schizophrenia Proneness Instrument-Adult version (SPI-A) O4 + F1 + F2 + F3 sum score across *DAOA* rs3916971 genotypes in genotype **(A)**, dominant **(B)**, and recessive **(C)** model at baseline and last-available follow-up time point until 36 months. Data are presented as mean ± SEM; ^#^0.008 < *p* < 0.05 (significant without Bonferroni correction).

**Figure 3:**
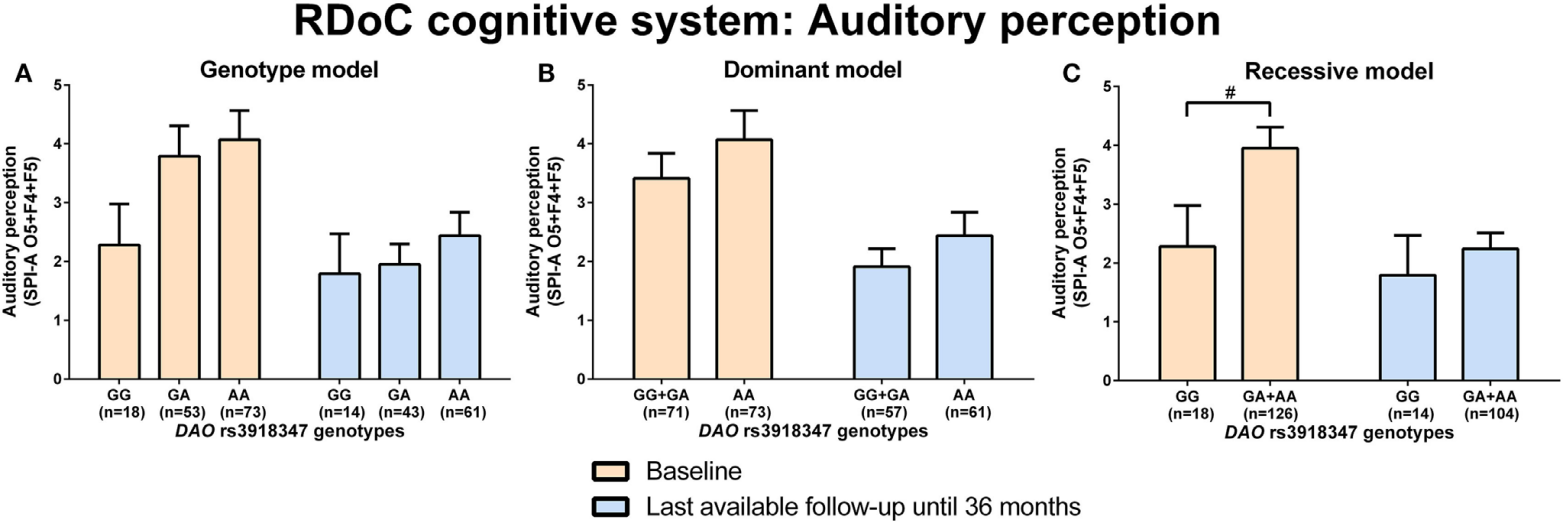
Differences in research domain criteria (RDoC) cognitive system auditory perception across d-amino acid oxidase activator (*DAO*) rs3918347 genotypes. Differences in Schizophrenia Proneness Instrument-Adult version (SPI-A) O5 + F4 + F5 sum score across *DAO* rs3918347 genotypes in genotype **(A)**, dominant **(B)**, and recessive **(C)** model at baseline and last-available follow-up time point until 36 months. Data are presented as mean ± SEM; ^#^0.008 < *p* < 0.05 (significant without Bonferroni correction).

d-amino acid oxidase activator rs746187 recessive (CC) genotype carriers experienced nominally more hopelessness (higher item 2 score, CDSS) than CT + TT genotype carriers at the last-available follow-up time point (LA) until 36 months (*p* = 0.04) analyzed by Mann–Whitney test (RDoC-negative valence system: loss; Figures 1A–C; power = 0.62; effect size = 0.55; Table S8 in Supplementary Material). *DAOA* rs3916971 recessive (TT) genotype carriers experienced nominally more disturbances in visual perception (higher sum of O4 + F1 + F2 + F3, SPI-A) than TC + CC genotype carriers at LA until 36 months (*p* = 0.009) analyzed by Mann–Whitney test (RDoC cognitive system: visual perception; Figures 2A–C; power = 0.28; effect size = 0.34; Table S8 in Supplementary Material). Individuals with *DAOA* rs3916971 TC + CC versus TT genotype improved and had fewer visual perceptual disturbances at LA until 36 months compared to baseline, but at LA until 36 months versus baseline, they continued to have less visual perceptual disturbances than individuals with TT genotype (Figure 2C). There were no significant differences in negative valence (threat; Table S5 in Supplementary Material; power range: 0.05–0.72, Table S8 in Supplementary Material), auditory perception disturbances (Table S6 in Supplementary Material; power range: 0.05–0.56, Table S8 in Supplementary Material), and cognitive control (Table S7 in Supplementary Material; power range: 0.05–0.31, Table S8 in Supplementary Material) in individuals with *DAOA* SNP genotypes (rs3916971, rs778293, rs746187).

d-amino acid oxidase rs3918347 GA + AA genotype carriers experienced nominally (*p* = 0.04) more disturbances in auditory perception (higher sum of O5 + F4 + F5, SPI-A) than GG genotype carriers at baseline analyzed by Mann–Whitney test (RDoC cognitive system: auditory perception; Figures 3A–C; power = 0.41; effect size = 0.44; Table S8 in Supplementary Material). Individuals with *DAO* rs3918347 GA + AA versus GG genotype improved and had lesser auditory perceptual disturbances at LA until 36 months compared to baseline, but at LA until 36 months versus baseline, they continued to experience more auditory perceptual disturbances than individuals with CC genotype (Figure 3C). We did not find significant differences in negative valence systems (anxiousness and hopelessness; Table S5 in Supplementary Material; power range: 0.05–0.92, Table S8 in Supplementary Material), visual perception disturbances (Table S6 in Supplementary Material; power range: 0.05–0.57, Table S8 in Supplementary Material), and cognitive control (Table S7 in Supplementary Material; power range: 0.05–0.22, Table S8 in Supplementary Material) in individuals with *DAO* (rs3918347, rs4623951) SNP genotypes.

**Figure 4:**
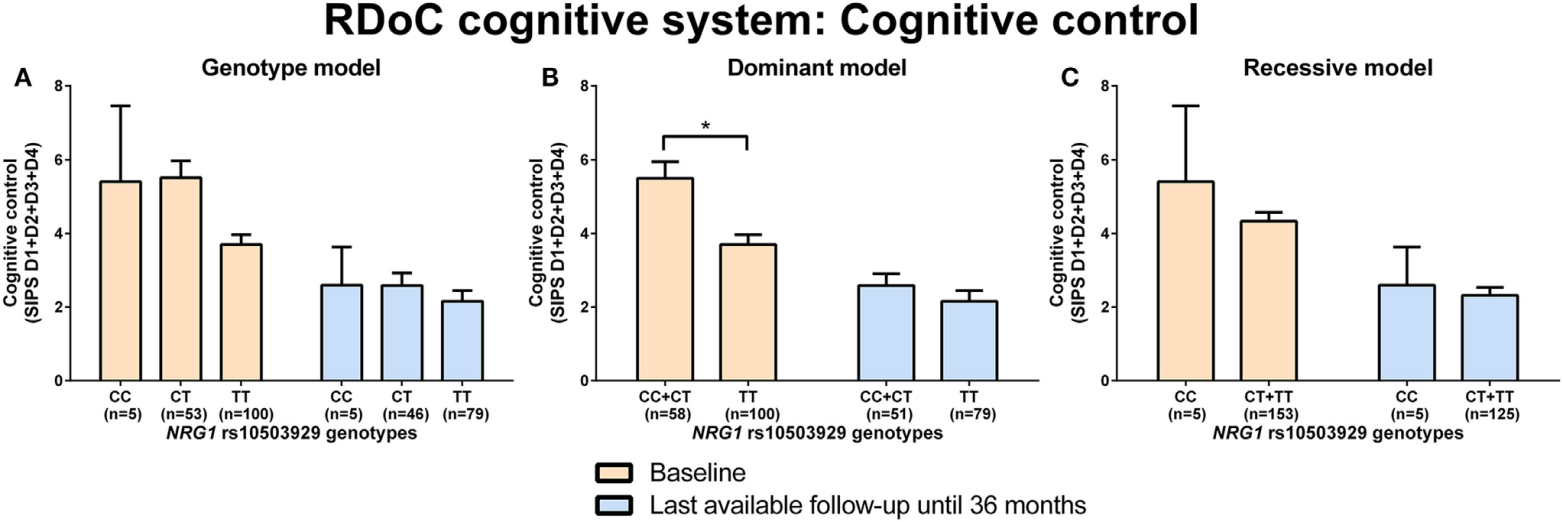
Differences in research domain criteria (RDoC) cognitive system cognitive control across neuregulin 1 (*NRG1*) rs10503929 genotypes. Differences in Structured Interview for Prodromal Syndromes (SIPS) sum score of D1-D4 across *NRG1* rs10503929 genotypes in genotype **(A)**, dominant **(B)**, and recessive **(C)** model at baseline and last-available follow-up time point until 36 months. Data are presented as mean ± SEM; *p < 0.008 (significant with Bonferroni correction).

Neuregulin 1 rs10503929 CC + CT genotype carriers had significantly (*p* = 0.001) more disorganized symptoms (higher sum of the D1–D4 score, SIPS) than TT genotype carriers at baseline analyzed by Mann–Whitney test (RDoC cognitive system: cognitive control; Figures 4A–C; power = 0.99; effect size = 0.59; Table S8 in Supplementary Material). There were no significant differences in negative valence systems (anxiousness and hopelessness; Table S5 in Supplementary Material; power range: 0.07–0.51, Table S8 in Supplementary Material) and visual and auditory perception disturbances (Table S6 in Supplementary Material; power range: 0.05–0.36, Table S8 in Supplementary Material) in individuals with *NRG1* (rs10503929) SNP genotypes.

To analyze the effect of age on RDoC domains across *DAO, DAOA*, and *NRG1* SNPs, we performed an analysis of covariance with age as a covariate after transformation of not normally distributed BAI, SPI-A, and SIPS data, as described previously (52). We did not find a significant effect of age on differences in RDoC domains across *DAO, DAOA*, and *NRG1* SNPs (data not shown).

### *NRG1, DAO*, and *DAOA* mRNA Expression across RDoC and Clinical Domains

There was a significant positive correlation between increased *NRG1* mRNA levels and higher scores on the RDoC-negative valence system loss at LA until 36 months (Table S9 in Supplementary Material). Apart from this, there were no significant correlations between *NRG1* mRNA levels and RDoC domains at baseline and at LA until 36 months (Table S9 in Supplementary Material). There were no statistically significant differences in *NRG1* mRNA levels across clinical phenotypes (Table S10 in Supplementary Material). We did not find significant differences in *NRG1* mRNA levels across *NRG1* rs10503929 SNP genotypes, dominant, and recessive models analyzed by Mann–Whitney and Kruskal–Wallis tests (Table S11 in Supplementary Material). We were unable to quantify *DAO* and *DAOA* mRNA levels in the whole blood of at-risk population.

## Discussion

In this 3-year follow-up study of 185 at-risk individuals, *NRG1* rs10503929 CC + CT versus TT genotype carriers experienced significantly more disorganized symptoms, *DAOA* rs746187 CC versus CT + TT genotype, *DAOA* rs3916971 TT versus TC + CC genotype, and *DAO* rs3918347 GA + AA versus GG genotype carriers experienced nominally more hopelessness, visual perception disturbances, and auditory perception disturbances, respectively. Moreover, we found no significant association between *DAO, DAOA, NRG1* SNPs, and conversion to schizophrenia spectrum disorders; however, we did find a nominally increased risk for APSS with the G-allele of *DAO* rs3918347 carriers.

The *DAO, DAOA*, and *NRG1* SNPs did not predict conversion to schizophrenia spectrum disorders at 36 months follow-up. This lack of association may be due to a low conversion rate of 14.6%, a high dropout rate of 50% at 36 months follow-up, and that the comparison group of non-converters was not a healthy control group but rather a heterogeneous group of HR and UHR individuals. In this heterogeneous at-risk population, individuals are likely to be on different developmental trajectories of/toward various neuropsychiatric disorders, which might have further complicated the genetic prediction of transition to schizophrenia in our study. Furthermore, our conversion group not only contained patients with schizophrenia but also patients with other schizophrenia spectrum disorders. Thus, recruiting a more homogeneous at-risk population has been more appropriate (53, 54). In contrast to previous meta-analyses showing conversion rates of 29–32% at 36 months follow-up (4, 6), our study had a low conversion rate of 14.6%. A study of 82 UHR individuals showed that 100% of the *DAOA* rs1341402 CC genotype carriers (*n* = 4) compared to 50% of the *DAOA* rs778294 AA genotype carriers (*n* = 10; A-allele protective against schizophrenia) progressed to psychosis within 24 months (13). However, a recent study with 225 UHR individuals did not replicate these findings (14). Furthermore, another study of 67 UHR individuals showed that 100% of TT genotype carriers (*n* = 25) of *NRG1* rs62510682 developed psychosis within 12-months (12), but this finding was not replicated in the aforementioned study with 225 UHR individuals (14). About 46% of the *NRG1* rs4281084 AA genotype UHR carriers (*n* = 13) and 44% of the T-allele UHR carriers (*n* = 45) transitioned to psychosis within a 15-year follow-up period (14). Thus, there is ambiguity regarding the association of *DAOA* and *NRG1* polymorphisms with the transition to psychosis. We did not assess the aforementioned *DAOA* and *NRG1* SNPs in this study as we only focused on the SNPs associated with schizophrenia (10, 43).

Our finding that *NRG1* rs10503929 TT genotype carriers had significantly less disorganized symptoms than the dominant (CC + CT) genotype carriers at baseline (RDoC cognitive control) is consistent with previous finding of a significant association of *NRG1* rs10503929 with cognitive domains (abstraction and mental flexibility, attention, and verbal memory) in schizophrenia patients, in which the C-allele (protective against schizophrenia) was associated with decreased cognitive performance (55). We found that *NRG1* rs10503929 (CT + TT) versus CC genotype carriers had nominally more auditory perception disturbances. To our knowledge, there are no previous studies on associations between the *NRG1* rs10503929 (exon 8/9/10) and perceptual disturbances. However, a study in adolescents demonstrated that *NRG1* rs3924999 (exon 2) was associated with perceptual disturbances (56).

The nominal associations between *DAOA* rs746187 and the RDoC-negative valence system: loss, and that between *DAOA* rs3916971 and the RDoC cognitive system: visual perception, points to the possible role of *DAOA* variations in modulating endophenotypes underlying psychosis risk. A recent study found a nominal association of *DAOA* rs3916971 with a psychotic disorder (57). Another study conducted in healthy male controls found that *DAOA* rs3916971 schizophrenia risk C-allele carriers had worse visual-spatial skills (58, 59). In our study, *DAO* rs3918347 GA + AA genotype carriers experienced nominally more auditory perception disturbances than GG genotype carriers at baseline (RDoC cognitive system). Another study found a negative association of *DAO* rs3918346 with neurocognitive functioning in schizophrenia patients (60). Therefore, the association of *DAO* and *DAOA* SNPs with hopelessness and perception disturbances of our study needs further confirmation.

We further found that *NRG1* mRNA levels increased with higher CDSS hopelessness scores. Previous postmortem studies have shown increased *NRG1* mRNA levels in the hippocampus (61) and prefrontal cortex (62, 63) of schizophrenia patients compared to that of healthy controls. Moreover, studies have shown dysfunctions in these regions might lead to hopelessness (64–66).

We examined associations of *DAO, DAOA*, and *NRG1* SNPs with risk profiles in individuals at risk for psychosis. For the sake of higher homogeneity, we also focused on the APSS subsample, a classification that the DSM-5 working group included under “conditions for further study” (67). We found that the schizophrenia risk G-allele of *DAO* rs3918347 had a tendency to be a risk allele for APSS compared to the remaining help-seeking group. This result has to be interpreted cautiously because the help-seeking group is not a healthy control group, but a mixed group of BLIPS, state-trait risk criterion, and HR.

We did not find any significant differences in *NRG1* mRNA levels between converters to schizophrenia spectrum disorders versus non-converters, and APSS versus all other help-seeking non-converters, which might be due to small subsample size leading to modest power (Tables S3 and S4 in Supplementary Material) and heterogeneous subgroups. Our results are in contrast to a study, which showed that *NRG1* (type I and II isoforms) mRNA expression was significantly lower in blood of UHR individuals who transitioned to psychosis (*n* = 31) compared to non-converters (*n* = 66) and controls (*n* = 50) (68). The discrepancy in the results of our study and the aforementioned study might also be due to the differences in isolation method, number of reference genes, stability of the reference genes, and normalization method used to normalize *NRG1* mRNA levels to reference genes. A study conducted on immortalized lymphocytes showed that there was no difference in *NRG1* mRNA levels between schizophrenia patients and healthy controls (69).

We were unable to detect *DAO* and *DAOA* mRNA using qRT-PCR in the whole blood of individuals at-risk for psychosis, which is in line with a study that used RNA sequencing to detect *DAO* and *DAOA* mRNA in healthy participants (70). As qRT-PCR can detect low copy number genes (71), undetectable *DAO* and *DAOA* mRNA levels might suggest either very low expression below the detection limit of qRT-PCR or extremely localized expression (44). The reasons for very low or no expression of *DAO* and *DAOA* mRNA in blood might be highly methylated (75–90%) Illumina CpG sites of *DAO* and *DAOA* genes in healthy individuals (72). Another reason for this low or no expression might be the expression of these genes specifically in the brain (47, 73) because of their role in glutamatergic neurotransmission via NMDA receptors (25).

In our study, *DAO* rs4623951 genotype data showed that the study population deviated from the HWE. Since we controlled for genotyping errors, this deviation from HWE might be due to the observed excess of CT heterozygotes (54%). This excess heterozygosity might be caused by “selection favoring heterozygotes, outbreeding, and negative assortative mating” (74). As deviation from the HWE creates bias in the associations reported (75), the association results of *DAO* rs4623951 SNP should be interpreted with caution. As this study did not have all the instruments suggested by RDoC for negative valence and cognitive systems, we used an exploratory approach, using the instruments available in this study to create the respective constructs. Thus, future studies are needed to confirm our RDoC findings.

Our study has several limitations, which must be acknowledged. Although a total of 185 individuals at risk for psychosis were recruited, sample sizes within genotypes across clinical and RDoC domains were small and the power of the study was modest. In this study, only a small percentage (14.6%) of at-risk individuals converted to schizophrenia at 36 months follow-up, 50% of individuals dropped out of the study before 36 months, and there was no healthy control group. The conversion status of the dropouts is unknown, and thus, it is not possible to reliably determine the conversion rate. The group of all other help seeking individuals was at heterogeneous risk (BLIPS, state-trait criteria, COPER, COGDIS). The sample size at 36-month follow-up for different psychopathology scales was small because of the high dropout rate. To circumvent this problem, we used the last psychopathology assessment available from each individual. Therefore, the results of LA until 36 months should be interpreted with caution, as they are not based on a homogeneous 36-month follow-up score. We studied only few genes (three genes) and a relatively small number of their polymorphisms (six SNPs), which might be the reason for not finding markers for predicting conversion to schizophrenia spectrum disorders. However, these genes and their polymorphisms are still of interest due to their importance in glutamatergic neurotransmission. This study also has strengths, which needs to be highlighted. We recruited individuals from a broad age range (13–35 years) and used age-specific scales (e.g., SPI-A/SPI-CY). Most of the published literature in at-risk population has focused on clinical phenotypes. This study used both clinical phenotypes and RDoC domains, especially negative valence and cognitive systems, due to the role of the studied genes in the glutamate hypothesis of schizophrenia.

In summary, although *DAO, DAOA*, and *NRG1* SNPs did not emerge as predictive markers for conversion to schizophrenia spectrum disorders, future association studies with larger cohorts, and longer follow-ups are needed to confirm the role of these genes in transition to schizophrenia spectrum disorders in the at-risk population. We also identified an association between the studied glutamatergic variants and RDoC-negative valence and cognitive systems, which indirectly implicates the role of these genetic variants in the glutamate hypothesis of schizophrenia. Future studies using RDoC domains might help to determine specific endophenotypes within at-risk populations. This might provide clinically useful, genetically informed risk prediction for dimensional and categorical outcomes among populations who maybe at-risk for developing psychosis.

## Ethics Statement

This study was approved by the Cantonal Ethics Commission of Zurich (Ref. Nr. EK: E-63/2009) and complies with the Declaration of Helsinki. Informed written consent was obtained from adult participants and legal guardians of minors, and written assent was obtained from minors.

## Author Contributions

WR, AT, KH, and SW designed the genetic part of the ZInEP study; AT, MG, SW, and EG designed the present genetic glutamatergic study. AT, MG, and MF collected the data and blood samples. VJ performed experiments, analyzed data, and drafted the manuscript. AT, MG, MF, KH, CUC, WR, SW, and EG reviewed the manuscript. All authors contributed to and have approved the final manuscript.

## Conflict of Interest Statement

CC has been a consultant and/or advisor to or has received honoraria from: Alkermes, Allergan, Bristol-Myers Squibb, Gerson Lehrman Group, IntraCellular Therapies, Janssen/J&J, LB Pharma, Lundbeck, Medavante, Medscape, Neurocrine, Otsuka, Pfizer, ProPhase, Sunovion, Takeda, and Teva. He has provided expert testimony for Bristol-Myers Squibb, Janssen, and Otsuka. He served on a Data Safety Monitoring Board for Lundbeck and Pfizer. He received grant support from Takeda. SW has received lecture honoraria from Eli-Lilly, Astra Zeneca, Shire, and Opopharma in the last 5 years. Outside professional activities and interests are declared under the link of the University of Zurich http://www.uzh.ch/prof/ssl-dir/interessenbindungen/client/web. WR received during the last five years consultant and lecture honoraria from Elli Lilly, Janssen-Cilag, Forum für Medizinische Fortbildung, Berufliches Bildungs-und Rehabilitationszentrum Wien, Schweizer Gesellschaft für Sportmedizin. The other authors declare that the research was conducted in the absence of any commercial or financial relationships that could be construed as a potential conflict of interest.

## Footnotes

^1^http://www.zinep.ch.

^2^https://www.medcalc.org/calc/odds_ratio.php.
