# Supplementary material for: Prediction Analysis for Transition to Schizophrenia in Individuals at Clinical High Risk for Psychosis: The Relationship of DAO, DAOA, and NRG1 Variants with Negative Symptoms and Cognitive Deficits
Source: Front Psychiatry. 2017 Dec 20;8:292. doi: 10.3389/fpsyt.2017.00292 (PMC5742321; doi:10.3389/fpsyt.2017.00292)
Supplement: Supplementary file 1 [file Presentation_1.PDF]

frontiers

Supplementary Material

Prediction analysis for transition to schizophrenia in individuals at clinical high-risk for psychosis: The relationship of *DAO*, *DAOA* and *NRG1* variants with negative symptoms and cognitive deficits

Vinita Jagannath[1#], Anastasia Theodoridou[2,3#], Miriam Gerstenberg[1#], Maurizia Franscini[1], Karsten Heekeren[2,3], Christoph U. Correll[4,5,6], Wulf Rössler[2], Edna Grünblatt[1,7,8#*], Susanne Walitza[1,7,8#]

*** Correspondence:** Edna Grünblatt: edna.gruenblatt@kjpd.uzh.ch

1 Supplementary Results

Supplementary table S1 Demographics and diagnostic characteristics of the study population

Categorical variables		Baseline (n=185)	
		N	%
Female		75	40.5
Male		110	59.5
Converters		27	14.6
Non-converters		65	35.1
APSS		98	53
All other help-seeking (n=87)	COPER	28	15.1
	COGDIS	7	3.8
	HR (COPER+COGDIS)	43	23.2
	BLIPS	4	2.2
	State-trait criteria	5	2.7

Continuous variables	Baseline (n=185)			Last available follow-up data until 36 months (n=151)		
	N	Range	Mean±SEM	N	Range	Mean±SEM
Age in years	185	13-35	20.51±0.42	151	13-35	20.04±0.23
Threat (sum of BAI scale)	169	0-57	19.6±0.88	139	0-84	12.29±1.07
CDSS: item 2	185	0-3	1.06±0.07	151	0-3	0.67±0.07
Visual perception (SPI-A O4+F1+F2+F3)	111	1-15	3.39±0.32	98	1-15	2.29±0.27
Auditory perception (SPI-A O5+F4+F5)	173	0-16	3.77±0.29	135	0-15	2.17±0.23
Cognitive control (SIPS: sum of D1-D4)	185	0-15	4.46±0.22	150	0-10	2.40±0.20

APSS: attenuated positive symptoms syndrome; COPER: cognitive-perceptive basic symptoms; COGDIS: cognitive disturbances; BLIPS: brief limited intermittent psychotic symptoms; HR: high risk criteria; BAI: Beck Anxiety Inventory; CDSS: Calgary Depression Rating scale for Schizophrenia; SPI-A: Schizophrenia Proneness Instrument-Adult version (≥ 18 years); SPI-CY: Schizophrenia Proneness Instrument-Child and Youth version (< 18 years); SIPS: Structured Interview for Prodromal Symptoms; SEM: standard error of the mean

Supplementary table S2 Hardy-Weinberg equilibrium and minor allele frequencies of *DAO*, *DAOA* and *NRG1* SNPs in the study population

Gene	SNP ID	Genotype	N	H-W p-value	Minor allele	MAF	HapMap/ NCBI CEU MAF
**D-amino acid oxidase (*DAO*)**	**rs3918347**	GG	24	0.09	G	0.3189	0.274
		GA	70				
		AA	91				
	**rs4623951**	CC	18	**0.04***	C	0.3676	0.412
		CT	100				
		TT	67				
**D-amino acid oxidase activator (*DAOA*)**	**rs3916971**	TT	42	0.14	T	0.4462	0.394
		TC	82				
		CC	62				
	**rs778293**	GG	43	0.18	G	0.4541	0.389
		GA	82				
		AA	60				
	**rs746187**	CC	26	0.87	C	0.3703	0.375
		CT	85				
		TT	74				
**Neuregulin 1 (*NRG1*)**	**rs10503929**	CC	7	0.82	C	0.207	0.225
		CT	63				
		TT	116				

H-W: Hardy-Weinberg; MAF: minor allele frequency; SNP: single nucleotide polymorphism; *p<0.05 (**bold font**)

Supplementary table S3 Association of *DAO*, *DAOA* and *NRG1* SNPs with converters to schizophrenia-spectrum disorders (n=27) versus non-converters (n=65) at 36 months follow-up and power analysis

Gene	SNP ID	Minor allele	Model	Genotype / allele	Phenotype		OR	95% CI	p-value	Power[b]
					Converters (N)	Non-converters (N)				
**D-amino acid oxidase (*DAO*)**	**rs3918347**	G	Genotypic	GG	2	6	-	-	0.27[a]	-
				GA	10	18				
				AA	8	35				
			Allelic	G	14	30	1.58	0.73-3.41	0.25	0.29
				A	26	88				
			Dominant	GG+GA	12	24	2.19	0.78-6.15	0.14	0.31
				AA	8	35				
			Recessive	GG	2	6	0.98	0.18-5.30	0.98	0.05
				GA+AA	18	53				
	**rs4623951**	C	Genotypic	CC	3	7	-	-	0.62[a]	-
				CT	9	34				
				TT	8	18				
			Allelic	C	15	48	0.88	0.41-1.83	0.72	0.06
				T	25	70				
			Dominant	CC+CT	12	41	0.66	0.23-1.89	0.44	0.13
				TT	8	18				
			Recessive	CC	3	7	1.31	0.30-5.64	0.72	0.08
				CT+TT	17	52				
**D-amino acid oxidase activator (*DAOA*)**	**rs3916971**	T	Genotypic	TT	6	11	-	-	0.20[a]	-
				TC	5	28				
				CC	9	20				
			Allelic	T	17	50	1.01	0.49-2.08	0.99	0.05
				C	23	68				
			Dominant	TT+TC	11	39	0.63	0.22-1.76	0.38	0.14
				CC	9	20				
			Recessive	TT	6	11	1.87	0.59-5.96	0.29	0.22
				TC+CC	14	48				
	**rs778293**	G	Genotypic	GG	3	14	-	-	0.68[a]	-
				GA	9	22				
				AA	8	22				
			Allelic	G	15	50	0.79	0.38-1.66	0.54	0.09
				A	25	66				
			Dominant	GG+GA	12	36	0.92	0.32-2.59	0.87	0.05
				AA	8	22				
			Recessive	GG	3	14	0.55	0.14-2.18	0.40	0.20
				GA+AA	17	44				
	**rs746187**	C	Genotypic	CC	3	12	-	-	0.69[a]	-
				CT	11	26				
				TT	6	21				
			Allelic	C	17	50	1.01	0.49-2.08	0.99	0.05
				T	23	68				
			Dominant	CC+CT	14	38	1.29	0.43-3.85	0.65	0.08
				TT	6	21				
			Recessive	CC	3	12	0.69	0.17-2.75	0.60	0.11
				CT+TT	17	47				
**Neuregulin 1 (*NRG1*)**	**rs10503929**	C	Genotypic	CC	0	1	-	-	0.74[a]	-
				CT	6	21				
				TT	14	37				
			Allelic	C	6	23	0.73	0.27-1.94	0.53	0.14
				T	34	95				
			Dominant	CC+CT	6	22	0.72	0.24-2.15	0.56	0.10
				TT	14	37				
			Recessive	CC	0	1	0.95	0.04-24.3	0.98	0.05
				CT+TT	20	58				

*p<0.008 (**bold font**); #p<0.05 (*italics*) (significant without Bonferroni correction); [a] Chi-square p-value; [b] power (1-β) calculated using the Fisher's exact test for two independent proportions; OR: Odds ratio; CI: confidence interval for the OR

Supplementary table S4 Association of *DAO*, *DAOA* and *NRG1* SNPs with APSS (n=98) compared to all other help-seeking group (n=87) at baseline and power analysis

Gene	SNP ID	Minor allele	Model	Genotype/ allele	Phenotype APSS (N)	Phenotype Help-seeking (N)	OR	95% CI	p-value	Power[b]
D-amino acid oxidase (*DAO*)	rs3918347	G	Genotypic	GG	11	5	-	-	0.053[a]	-
				GA	37	26				
				AA	33	45				
			Allelic	G	59	36	1.84	1.13-3.01	*0.01#*	0.76
				A	103	116				
			Dominant	GG+GA	48	31	2.11	1.12-3.99	*0.02#*	0.63
				AA	33	45				
			Recessive	GG	11	5	2.23	0.74-6.75	0.16	0.69
				GA+AA	70	71				
	rs4623951	C	Genotypic	CC	7	7	-	-	0.99[a]	-
				CT	43	40				
				TT	31	29				
			Allelic	C	57	54	0.99	0.62-1.57	0.95	0.05
				T	105	98				
			Dominant	CC+CT	50	47	1.00	0.52-1.90	0.99	0.05
				TT	31	29				
			Recessive	CC	7	7	0.93	0.31-2.80	0.90	0.06
				CT+TT	74	69				
D-amino acid oxidase activator (*DAOA*)	rs3916971	T	Genotypic	TT	17	19	-	-	0.79[a]	-
				TC	33	32				
				CC	31	26				
			Allelic	T	67	70	0.85	0.54-1.32	0.46	0.11
				C	95	84				
			Dominant	TT+TC	50	51	0.82	0.43-1.58	0.56	0.09
				CC	31	26				
			Recessive	TT	17	19	0.82	0.39-1.71	0.58	0.09
				TC+CC	64	58				
	rs778293	G	Genotypic	GG	17	17	-	-	0.88[a]	-
				GA	34	35				
				AA	29	25				
			Allelic	G	68	69	0.91	0.58-1.42	0.68	0.07
				A	92	85				
			Dominant	GG+GA	51	52	0.85	0.44-1.64	0.62	0.09
				AA	29	25				
			Recessive	GG	17	17	0.95	0.45-2.04	0.90	0.05
				GA+AA	63	60				
	rs746187	C	Genotypic	CC	10	14	-	-	0.57[a]	-
				CT	39	34				
				TT	32	28				
			Allelic	C	59	62	0.83	0.53-1.31	0.43	0.13
				T	103	90				
			Dominant	CC+CT	49	48	0.89	0.47-1.70	0.73	0.07
				TT	32	28				
			Recessive	CC	10	14	0.62	0.26-1.50	0.29	0.33
				CT+TT	71	62				
Neuregulin 1 (*NRG1*)	rs10503929	C	Genotypic	CC	3	1	-	-	0.19[a]	-
				CT	33	23				
				TT	45	53				
			Allelic	C	39	25	1.64	0.93-2.86	0.08	0.60
				T	123	129				
			Dominant	CC+CT	36	24	1.77	0.92-3.39	0.09	0.42
				TT	45	53				
			Recessive	CC	3	1	2.92	0.30-28.7	0.36	0.89
				CT+TT	78	76				

*p<0.008 (**bold font**); #p<0.05 (*italics*) (significant without Bonferroni correction); [a] Chi-square p-value; [b] power (1-β) calculated using the Fisher's exact test for two independent proportions; APSS: attenuated positive symptoms syndrome; OR: Odds ratio; CI: confidence interval for the OR

Supplementary table S5 Differences in RDoC domain negative valence systems: threat (acute and sustained) and loss across *DAO*, *DAOA* and *NRG1* SNPs

Gene	SNP ID	Model	Genotypes	Negative valence systems											
				Threat (sum of BAI scale)						Loss (CDSS: item 2)					
				Baseline			LA			Baseline			LA		
				N	Mean± SEM	p-value[a]	N	Mean± SEM	p-value[a]	N	Mean± SEM	p-value[a]	N	Mean± SEM	p-value[a]
D-amino acid oxidase (*DAO*)	rs3918347	G	GG	16	16.3±1.8		12	15.8±6.9		18	1.1±0.2		14	0.64±0.3	
			GA	57	19.4±1.5	0.48	46	11.8±1.5	0.84	59	0.97±0.1	0.47	48	0.69±0.1	0.73
			AA	67	20.7±1.5		62	11.8±1.5		78	1.1±0.1		67	0.64±0.1	
		D	GG+GA	73	18.7±1.2	0.42	58	12.7±1.8	0.71	77	0.99±0.1	0.34	62	0.68±0.1	0.62
			AA	67	20.7±1.5		62	11.8±1.5		78	1.1±0.1		67	0.64±0.1	
		R	GG	16	16.3±1.8	0.26	12	15.8±6.9	0.77	18	1.1±0.2	0.72	14	0.64±0.3	0.69
			GA+AA	124	20.1±1.1		108	11.8±1.1		137	1.1±0.1		115	0.66±0.1	
	rs4623951	G	CC	13	24.0±3.6		15	13.5±3.4		15	1.1±0.1		15	0.67±0.2	
			CT	76	20.2±1.3	0.23	64	13.6±1.9	0.50	81	1.2±0.1	0.45	69	0.65±0.1	0.95
			TT	51	17.7±1.5		41	9.5±1.3		59	0.92±0.1		45	0.67±0.1	
		D	CC+CT	89	20.8±1.3	0.16	79	13.6±1.6	0.24	96	1.2±0.1	0.21	84	0.65±0.1	0.77
			TT	51	17.7±1.5		41	9.5±1.3		59	0.92±0.1		45	0.67±0.1	
		R	CC	13	24.0±3.6	0.19	15	13.5±3.4	0.74	15	1.1±0.1	0.62	15	0.67±0.2	0.97
			CT+TT	127	19.2±1.0		105	12.0±1.3		140	1.1±0.1		114	0.66±0.1	
D-amino acid oxidase activator (*DAOA*)	rs3916971	G	TT	31	20.5±2.0		24	17.3±4.0		36	1.1±0.2		31	0.61±0.2	
			TC	62	20.0±1.4	0.68	50	11.4±1.6	0.47	67	0.97±0.1	0.63	52	0.60±0.1	0.65
			CC	47	18.7±1.8		46	10.4±1.3		52	1.2±0.1		46	0.76±0.1	
		D	TT+TC	93	20.2±1.1	0.38	74	13.3±1.7	0.69	103	1.0±0.1	0.40	83	0.60±0.1	0.37
			CC	47	18.7±1.8		46	10.4±1.3		52	1.2±0.1		46	0.76±0.1	
		R	TT	31	20.5±2.0	0.78	24	17.3±4.0	0.22	36	1.1±0.2	0.93	31	0.61±0.2	0.87
			TC+CC	109	19.4±1.1		96	10.9±1.0		119	1.1±0.1		98	0.67±0.1	
	rs778293	G	GG	32	20.9±2.2		24	18.1±3.9		35	1.1±0.2		28	0.89±0.2	
			GA	60	19.1±1.3	0.82	49	10.4±1.5	0.21	68	0.94±0.1	0.35	53	0.51±0.1	0.28
			AA	48	19.6±1.8		47	11.0±1.5		52	1.2±0.1		48	0.69±0.1	
		D	GG+GA	92	19.7±1.2	0.86	73	13.0±1.7	0.66	103	0.99±0.1	0.25	81	0.64±0.1	0.60
			AA	48	19.6±1.8		47	11.0±1.5		52	1.2±0.1		48	0.69±0.1	
		R	GG	32	20.9±2.2	0.54	24	18.1±3.9	0.08	35	1.1±0.2	0.71	28	0.89±0.2	0.24
			GA+AA	108	20.9±2.2		96	10.7±1.1		120	1.1±0.1		101	0.59±0.1	
	rs746187	G	CC	21	19.9±3.2		20	10.7±2.2		24	1.4±0.2		21	1.05±0.2	
			CT	64	21.3±1.3	0.11	56	13.6±1.9	0.38	72	0.88±0.1	*0.04*#	61	0.54±0.1	0.11
			TT	55	17.7±1.5		44	11.2±1.9		59	1.2±0.1		47	0.64±0.1	
		D	CC+CT	85	20.9±1.3	0.07	76	12.8±1.5	0.22	96	1.0±0.1	0.40	82	0.67±0.1	0.91
			TT	55	17.7±1.5		44	11.2±1.9		59	1.2±0.1		47	0.64±0.1	
		R	CC	21	19.9±3.2	0.68	20	10.7±2.2	0.86	24	1.4±0.2	0.052	21	1.05±0.2	*0.04*#
			CT+TT	119	19.6±1.0		100	12.5±1.3		131	1.0±0.1		108	0.58±0.1	
Neuregulin 1 (*NRG1*)	rs10503929	G	CC	5	14.0±5.6		4	16.5±7.3		5	0.6±0.4		5	0.80±0.6	
			CT	46	20.9±1.8	0.47	42	13.4±1.9	0.53	51	1.2±0.1	0.37	44	0.55±0.1	0.82
			TT	89	19.3±1.2		74	11.3±1.5		99	1.0±0.1		80	0.71±0.1	
		D	CC+CT	51	20.2±1.7	0.81	46	13.7±1.8	0.31	56	1.1±0.1	0.65	49	0.57±0.1	0.54
			TT	89	19.3±1.2		74	11.3±1.5		99	1.0±0.1		80	0.71±0.1	
		R	CC	5	14.0±5.6	0.27	4	16.5±7.3	0.49	5	0.6±0.4	0.23	5	0.80±0.6	0.99
			CT+TT	135	19.9±1.0		116	12.1±1.2		150	1.1±0.1		124	0.65±0.1	

[a] p-value by Kruskal-Wallis test for genotypic model and Mann-Whitney test for dominant and recessive models; *p<0.008 (**bold font**); # 0.008<p<0.05 (*italics*) (significant without Bonferroni correction); G: genotypic model; D: dominant model; R: recessive model; LA: last-available follow-up data until 36 months; BAI: Beck Anxiety Inventory; CDSS: Calgary Depression Rating scale for Schizophrenia; SEM: standard error of the mean

Supplementary table S6 Differences in RDoC domain cognitive systems: visual and auditory perception across *DAO*, *DAOA* and *NRG1* SNPs

Gene	SNP ID	Model	Genotypes	Cognitive systems											
				Visual perception (SPI-A O4+F1+F2+F3)						Auditory perception (SPI-A O5+F4+F5)					
				Baseline			LA			Baseline			LA		
				N	Mean± SEM	p-value[a]	N	Mean± SEM	p-value[a]	N	Mean± SEM	p-value[a]	N	Mean± SEM	p-value[a]
D-amino acid oxidase (*DAO*)	rs3918347	G	GG	9	3.1±1.4	0.40	8	1.1±0.1	0.31	18	2.3±0.7	0.13	14	1.8±0.7	0.34
			GA	37	3.6±0.5		34	2.4±0.4		52	3.8±0.5		42	2.0±0.4	
			AA	45	3.5±0.6		43	2.3±0.5		72	4.1±0.5		60	2.5±0.4	
		D	GG+GA	46	3.5±0.5	0.57	42	2.1±0.3	0.54	70	3.4±0.4	0.33	56	1.9±0.3	0.27
			AA	45	3.5±0.6		43	2.3±0.5		72	4.1±0.5		60	2.5±0.4	
		R	GG	9	3.1±1.4	0.32	8	1.1±0.1	0.26	18	2.3±0.7	*0.04*[#]	14	1.8±0.7	0.19
			GA+AA	82	3.6±0.4		77	2.4±0.3		124	4.0±0.4		102	2.3±0.3	
	rs4623951	G	CC	8	5.3±1.3	0.15	9	4.1±1.7	0.30	13	5.2±1.1	0.15	13	3.0±0.9	0.20
			CT	50	3.4±0.5		49	2.2±0.4		76	3.6±0.4		65	2.3±0.3	
			TT	33	3.3±0.6		27	1.7±0.3		53	3.7±0.6		38	1.8±0.4	
		D	CC+CT	58	3.7±0.5	0.44	58	2.5±0.4	0.48	89	3.8±0.4	0.18	78	2.4±0.3	0.13
			TT	33	3.3±0.6		27	1.7±0.3		53	3.7±0.6		38	1.8±0.4	
		R	CC	8	5.3±1.3	0.053	9	4.1±1.7	0.13	13	5.2±1.1	0.09	13	3.0±0.9	0.19
			CT+TT	83	3.4±0.4		76	2.0±0.3		129	3.6±0.3		103	2.1±0.3	
D-amino acid oxidase activator (*DAOA*)	rs3916971	G	TT	26	4.1±0.8	0.09	22	2.9±0.6	*0.02*[#]	32	3.6±0.5	0.36	26	1.8±0.4	0.96
			TC	35	2.2±0.3		32	1.9±0.5		61	4.2±0.6		46	2.5±0.5	
			CC	30	4.6±0.8		31	2.1±0.5		49	3.3±0.6		44	2.1±0.3	
		D	TT+TC	61	3.0±0.4	0.17	54	2.3±0.4	0.73	93	4.0±0.4	0.15	72	2.3±0.4	0.77
			CC	30	4.6±0.8		31	2.1±0.5		49	3.3±0.6		44	2.1±0.3	
		R	TT	26	4.1±0.8	0.36	22	2.9±0.6	*0.009*[#]	32	3.6±0.5	0.55	26	1.8±0.4	0.95
			TC+CC	65	3.3±0.4		63	2.0±0.4		110	3.8±0.4		90	2.3±0.3	
	rs778293	G	GG	25	3.9±0.8	0.21	17	1.9±0.4	0.18	32	3.3±0.6	0.93	22	2.1±0.5	0.77
			GA	39	2.9±0.5		35	2.8±0.6		61	3.7±0.5		47	2.0±0.4	
			AA	27	4.0±0.7		33	1.8±0.4		49	4.1±0.6		47	2.5±0.5	
		D	GG+GA	64	3.3±0.4	0.17	52	2.5±0.4	0.07	93	3.6±0.4	0.75	69	2.0±0.3	0.54
			AA	27	4.0±0.7		33	1.8±0.4		49	4.1±0.6		47	2.5±0.5	
		R	GG	25	3.9±0.8	0.62	17	1.9±0.4	0.49	32	3.3±0.6	0.76	22	2.1±0.5	0.91
			GA+AA	66	3.4±0.4		68	2.3±0.4		110	3.9±0.4		94	2.2±0.3	
	rs746187	G	CC	15	2.1±0.6	0.15	15	2.3±0.9	0.95	22	4.2±1.0	0.37	20	3.4±1.0	0.26
			CT	38	3.6±0.6		36	2.0±0.3		67	3.3±0.4		52	2.0±0.3	
			TT	38	4.0±0.6		34	2.4±0.6		53	4.2±0.6		44	2.0±0.3	
		D	CC+CT	53	3.2±0.5	0.27	51	2.1±0.4	0.74	89	3.5±0.4	0.32	72	2.3±0.4	0.89
			TT	38	4.0±0.6		34	2.4±0.6		53	4.2±0.6		44	2.0±0.3	
		R	CC	15	2.1±0.6	0.06	15	2.3±0.9	0.87	22	4.2±1.0	0.55	20	3.4±1.0	0.12
			CT+TT	76	3.8±0.4		70	2.2±0.3		120	3.7±0.4		96	2.0±0.2	
Neuregulin 1 (*NRG1*)	rs10503929	G	CC	2	2.5±1.5	0.64	2	1.0±0.0	0.20	5	1.0±0.5	0.17	4	0.8±0.3	0.30
			CT	28	3.8±0.6		28	2.3±0.4		44	3.8±0.5		41	2.5±0.4	
			TT	61	3.4±0.5		55	2.3±0.4		93	3.9±0.4		71	2.1±0.3	
		D	CC+CT	30	3.7±0.6	0.40	30	2.2±0.4	0.16	49	3.5±0.5	0.80	45	2.4±0.4	0.69
			TT	61	3.4±0.5		55	2.3±0.4		93	3.9±0.4		71	2.1±0.3	
		R	CC	2	2.5±1.5	0.81	2	1.0±0.0	0.40	5	1.0±0.5	0.06	4	0.8±0.3	0.17
			CT+TT	89	3.6±0.4		83	2.3±0.3		137	3.9±0.3		112	2.3±0.3	

[a] p-value by Kruskal-Wallis test for genotypic model and Mann-Whitney test for dominant and recessive models; *p<0.008 (**bold font**); # 0.008<p<0.05 (*italics*) (significant without Bonferroni correction); G: genotypic model; D: dominant model; R: recessive model; LA: last-available follow-up data until 36 months; SPI-A: Schizophrenia Proneness Instrument-Adult version (≥ 18 years); SPI-CY: Schizophrenia Proneness Instrument-Child and Youth version (< 18 years); SEM: standard error of the mean

Supplementary table S7 Differences in RDoC domain cognitive system: cognitive control across *DAO*, *DAOA* and *NRG1* SNPs

Gene	SNP ID	Model	Genotypes	Cognitive systems					
				Cognitive control (SIPS: sum of D1-D4)					
				Baseline			LA		
				N	Mean±SEM	p-value[a]	N	Mean±SEM	p-value[a]
D-amino acid oxidase (*DAO*)	rs3918347	G	GG	18	4.8±0.9	0.59	14	2.2±0.5	0.95
			GA	58	4.5±0.4		49	2.4±0.4	
			AA	79	4.1±0.3		65	2.2±0.3	
		D	GG+GA	76	4.6±0.4	0.32	63	2.3±0.3	0.75
			AA	79	4.1±0.3		65	2.2±0.3	
		R	GG	18	4.8±0.9	0.90	14	2.2±0.5	0.85
			GA+AA	137	4.3±0.2		114	2.3±0.2	
	rs4623951	G	CC	15	3.9±0.9	0.65	15	1.9±0.6	0.52
			CT	81	4.4±0.3		69	2.6±0.3	
			TT	59	4.4±0.4		44	2.0±0.3	
		D	CC+CT	96	4.3±0.3	0.56	84	2.4±0.3	0.73
			TT	59	4.4±0.4		44	2.0±0.3	
		R	CC	15	3.9±0.9	0.40	15	1.9±0.6	0.34
			CT+TT	140	4.4±0.3		113	2.3±0.2	
D-amino acid oxidase activator (*DAOA*)	rs3916971	G	TT	36	3.7±0.5	0.43	29	2.6±0.5	0.70
			TC	67	4.6±0.4		53	2.1±0.3	
			CC	52	4.4±0.4		46	2.4±0.4	
		D	TT+TC	103	4.3±0.3	0.67	82	2.2±0.3	0.91
			CC	52	4.4±0.4		46	2.4±0.4	
		R	TT	36	3.7±0.5	0.19	29	2.6±0.5	0.41
			TC+CC	119	4.5±0.3		99	2.2±0.2	
	rs778293	G	GG	35	3.8±0.5	0.49	28	2.7±0.5	0.32
			GA	68	4.7±0.4		52	2.4±0.4	
			AA	52	4.2±0.4		48	1.9±0.3	
		D	GG+GA	103	4.4±0.3	0.90	80	2.5±0.3	0.19
			AA	52	4.2±0.4		48	1.9±0.3	
		R	GG	35	3.8±0.5	0.25	28	2.7±0.5	0.22
			GA+AA	120	4.5±0.3		100	2.2±0.2	
	rs746187	G	CC	24	3.4±0.6	0.22	21	3.0±0.6	0.61
			CT	73	4.6±0.4		59	2.1±0.3	
			TT	58	4.3±0.4		48	2.2±0.4	
		D	CC+CT	97	4.3±0.3	0.60	80	2.4±0.3	0.45
			TT	58	4.3±0.4		48	2.2±0.4	
		R	CC	24	3.4±0.6	0.08	21	3.0±0.6	0.38
			CT+TT	131	4.5±0.3		107	2.2±0.2	
Neuregulin 1 (*NRG1*)	rs10503929	G	CC	5	5.4±2.1	**0.003***	5	2.6±1.0	0.33
			CT	50	5.5±0.5		44	2.5±0.3	
			TT	100	3.7±0.3		79	2.2±0.3	
		D	CC+CT	55	5.5±0.5	**0.001***	49	2.5±0.3	0.14
			TT	100	3.7±0.3		79	2.2±0.3	
		R	CC	5	5.4±2.1	0.71	5	2.6±1.0	0.67
			CT+TT	150	4.3±0.2		123	2.3±0.2	

[a] p-value by Kruskal-Wallis test for genotypic model and Mann-Whitney test for dominant and recessive models; *p<0.008 (**bold font**); # 0.008<p<0.05 (*italics*) (significant without Bonferroni correction); G: genotypic model; D: dominant model; R: recessive model; LA: last-available follow-up data until 36 months; SIPS: Structured Interview for Prodromal Symptoms; SEM: standard error of the mean

Supplementary table S8 Power analysis for RDoC negative valence and cognitive systems across *DAO*, *DAOA*, and *NRG1* SNPs

Gene	SNP ID	Model	Negative valence systems								Cognitive systems											
			Threat (sum of BAI scale)				Loss (CDSS: item 2)				Visual perception (SPI-A O4+F1+F2+F3)				Auditory perception (SPI-A O5+F4+F5)				Cognitive control (SIPS: sum of D1-D4)			
			Baseline		LA		Baseline		LA		Baseline		LA		Baseline		LA		Baseline		LA	
			ES[a]	P[a]	ES[a]	P[a]	ES[a]	P[a]	ES[a]	P[a]	ES[a]	P[a]	ES[a]	P[a]	ES[a]	P[a]	ES[a]	P[a]	ES[a]	P[a]	ES[a]	P[a]
**D-amino acid oxidase (*DAO*)**	**rs3918347**	Genotypic	0.12	0.22	0.09	0.13	0.08	0.14	0.03	0.06	0.04	0.06	0.13	0.16	0.15	0.33	0.10	0.14	0.08	0.13	0.03	0.06
		Dominant	0.18	0.18	0.61	0.92	0.16	0.16	0.05	0.06	0.003	0.05	0.07	0.06	0.17	0.17	0.20	0.18	0.14	0.14	0.03	0.05
		Recessive	0.33	0.23	0.31	0.18	0.00	0.05	0.02	0.05	0.13	0.07	0.43	0.21	0.44	0.41	0.17	0.09	0.18	0.11	0.04	0.05
	**rs4623951**	Genotypic	0.21	0.76	0.15	0.29	0.12	0.25	0.01	0.05	0.16	0.24	0.24	0.49	0.11	0.20	0.12	0.20	0.05	0.08	0.12	0.22
		Dominant	0.26	0.32	0.32	0.38	0.26	0.34	0.02	0.05	0.11	0.08	0.26	0.19	0.02	0.05	0.20	0.17	0.04	0.06	0.18	0.16
		Recessive	0.40	0.78	0.11	0.07	0.09	0.06	0.01	0.05	0.55	0.31	0.76	0.57	0.38	0.26	0.32	0.19	0.17	0.10	0.20	0.11
**D-amino acid oxidase activator (*DAOA*)**	**rs3916971**	Genotypic	0.06	0.09	0.20	0.49	0.10	0.17	0.09	0.13	0.31	0.75	0.15	0.22	0.11	0.18	0.09	0.13	0.13	0.26	0.09	0.13
		Dominant	0.13	0.11	0.23	0.22	0.19	0.20	0.18	0.17	0.45	0.52	0.07	0.06	0.18	0.18	0.05	0.06	0.03	0.05	0.05	0.06
		Recessive	0.09	0.07	0.50	0.59	0.00	0.05	0.07	0.06	0.22	0.16	0.34	0.28	0.05	0.06	0.17	0.12	0.29	0.33	0.16	0.12
	**rs778293**	Genotypic	0.06	0.09	0.23	0.61	0.12	0.25	0.17	0.37	0.15	0.23	0.18	0.28	0.08	0.11	0.08	0.11	0.12	0.25	0.12	0.22
		Dominant	0.006	0.05	0.15	0.12	0.22	0.25	0.06	0.06	0.21	0.15	0.28	0.23	0.14	0.12	0.16	0.13	0.07	0.07	0.24	0.26
		Recessive	0.14	0.11	0.58	0.72	0.04	0.06	0.35	0.37	0.16	0.10	0.13	0.08	0.14	0.11	0.03	0.05	0.23	0.22	0.20	0.16
	**rs746187**	Genotypic	0.14	0.31	0.10	0.15	0.21	0.62	0.21	0.54	0.19	0.32	0.07	0.08	0.12	0.21	0.19	0.42	0.14	0.31	0.12	0.21
		Dominant	0.28	0.37	0.12	0.10	0.17	0.17	0.03	0.05	0.22	0.17	0.12	0.08	0.17	0.17	0.13	0.11	0.003	0.05	0.07	0.06
		Recessive	0.02	0.05	0.15	0.09	0.43	0.48	0.55	0.62	0.49	0.40	0.01	0.05	0.13	0.09	0.51	0.56	0.12	0.08	0.33	0.27
**Neuregulin 1 (*NRG1*)**	**rs10503929**	Genotypic	0.11	0.20	0.10	0.14	0.12	0.26	0.09	0.14	0.06	0.08	0.07	0.08	0.13	0.28	0.13	0.20	0.29	0.91	0.07	0.09
		Dominant	0.08	0.07	0.18	0.16	0.12	0.11	0.16	0.14	0.07	0.06	0.04	0.05	0.10	0.08	0.10	0.08	0.59	0.99	0.13	0.11
		Recessive	0.51	0.20	0.34	0.10	0.52	0.21	0.17	0.07	0.30	0.07	0.45	0.09	0.73	0.36	0.55	0.19	0.36	0.12	0.13	0.06

[a] ANOVA (for 3 groups) and t-test for 2 groups; LA: last-available follow-up data until 24 months; BAI: Beck Anxiety Inventory; CDSS: Calgary Depression Rating scale for Schizophrenia; SPI-A: Schizophrenia Proneness Instrument-Adult version (≥ 18 years); SPI-CY: Schizophrenia Proneness Instrument-Child and Youth version (< 18 years); SIPS: Structured Interview for Prodromal Symptoms; ES: effect size; P: Power; SEM: standard error of the mean

Supplementary table S9 Correlation between *NRG1* gene expression and RDoC domains at baseline and 36 months

RDoC domains	RDoC construct (scale)	Time-point	*NRG1* gene expression		
			N	Spearman's rank correlation co-efficient	p-value
**Negative valence systems**	**Threat (sum of BAI scale)**	**Baseline**	168	0.133	0.085
		**LA**	135	0.049	0.575
	**Loss (CDSS: item 2)**	**Baseline**	181	-0.057	0.444
		**LA**	146	0.196	**0.018***
**Cognitive systems**	**Visual perception (SPI-A O4+F1+F2+F3)**	**Baseline**	114	-0.012	0.901
		**LA**	103	-0.099	0.318
	**Auditory perception (SPI-A O5+F4+F5)**	**Baseline**	167	0.004	0.963
		**LA**	132	-0.046	0.598
	**Cognitive control (SIPS: sum of D1-D4)**	**Baseline**	182	0.051	0.491
		**LA**	145	0.106	0.203

*p<0.05 (**bold font**); LA: last-available follow-up data until 36 months; RDoC: Research Domain Criteria; BAI: Beck Anxiety Inventory; CDSS: Calgary Depression Rating scale for Schizophrenia; SPI-A: Schizophrenia Proneness Instrument-Adult version (≥18 years); SPI-CY: Schizophrenia Proneness Instrument-Child and Youth version (<18 years); SIPS: Structured Interview for Prodromal Symptoms

Supplementary table S10 *NRG1* gene expression in peripheral whole blood across different phenotypes and power analysis

Clinical phenotypes	*NRG1* gene expression			Effect size[a]	Power[a]
	N	Mean±SEM	Mann-Whitney p-value		
**Converters**	23	1.41±0.27	0.892	0.16	0.10
**Non-converters**	58	1.25±0.11			
**APSS**	80	1.36±0.12	0.650	0.16	0.17
**All other help-seeking**	76	1.21±0.09			

*p<0.05 (**bold font**); [a] t-test; APSS: attenuated positive symptoms syndrome; HR: high risk for psychosis; UHR: ultra-high risk for psychosis

Supplementary table S11 *NRG1* gene expression in peripheral whole blood across *NRG1* SNP genotypes and models

NRG1 SNP ID	*NRG1* mRNA expression											
	Genotypes	N	Mean± SEM	K-W p-value	Dom model	N	Mean± SEM	M-W p-value	Rec model	N	Mean± SEM	M-W p-value
**rs10503929**	CC	7	0.99± 0.15	0.975	CC+ CT	67	1.25± 0.12	0.848	CC	7	0.99± 0.15	0.868
	CT	60	1.28± 0.14		TT	105	1.19± 0.07		CT+ TT	165	1.22± 0.07	
	TT	105	1.19± 0.07									

*p<0.05 (**bold font**); K-W p-value: Kruskal-Wallis p-value; M-W p-value: Mann-Whitney p-value; Dom model: dominant model; Rec model: recessive model.
